# Bacteriological assessment of the hospital environment in two referral hospitals in Yaoundé-Cameroon

**DOI:** 10.11604/pamj.2015.20.224.4433

**Published:** 2015-03-12

**Authors:** Kamga Hortense Gonsu, Etienne Guenou, Michel Toukam, Valantine Ngum Ndze, Calixte Didier Mbakop, Dongmo Norbert Tankeu, Francois Xavier Mbopi-Keou, Samuel Takongmo

**Affiliations:** 1 Faculty of Medicine and Biomedical Sciences, University of Yaoundé I, Yaoundé, Cameroon; 2 School of Health Sciences, Catholic University of Central Africa, Yaoundé, Cameroon; 3 Biotechnology Center, University of Yaoundé I, Yaoundé, Cameroon

**Keywords:** Hospital environnement, nosocomial infection, reservoir, surgery, Cameroon

## Abstract

**Introduction:**

Many studies still show significant numbers of surgical patients contracting nosocomial infections each year globally with high morbidity and mortality. The aim of this study was to identify potential bacteria reservoirs that may be responsible for nosocomial infection in surgical services in the Yaoundé University Teaching Hospital (YUTH) and the Central Hospital Yaoundé (CHY).

**Methods:**

A cross sectional descriptive study was conducted from June to August 2012. Air, water, and surface samples were collected from two surgical services and subjected to standard bacteriological analysis.

**Results:**

A total of 143 surface samples were collected. Bacteria were isolated in all surfaces except from one trolley sample and a surgical cabinet sample. The predominant species in all services was coagulase negative Staphylococcus (CNS). The average number of colonies was 132. 82CFU/25cm^2^. The bacteria isolated in the air were similar to those isolated from surfaces. From the 16 water samples cultured, an average of 50.93 CFU/100ml bacteria were isolated. The distribution of isolated species showed a predominance of *Burkholderia cepacia*.

**Conclusion:**

These results showed the importance of the hospital environment as a potential reservoir and source of nosocomial infections amongst surgical patient at YUTH and CHY, thus we suggest that Public health policy makers in Cameroon must define, publish guidelines and recommendations for monitoring environmental microbiota in health facilities.

## Introduction

Despite significant scientific progress in the field of surgery, anesthesia and the use of antibiotics, estimates showed that an average of 2.5% of the 6 million surgical patients visiting US health facilities each year contract a nosocomial infection [1]. These lead to the increased burden on both the patient and society [1–4]. Nowadays, the assessment of health facilities environment (air, water, surfaces) has become part of a good health care quality and safety policy, including the assessment of risk of infection in surgery where surgical acts are performed sometimes with high-tech tools [5]. The main objective of this study was to identify potential bacteria reservoirs that may be responsible for nosocomial infection amongst surgical patient at the Yaoundé University Teaching hospital and at the Central Hospital Yaoundé in Cameroon. Specifically, the bacteriological quality of the air, water and surfaces in these health facilities was assessed.

## Methods

A cross sectional descriptive study was conducted from June to August 2012 in the surgical services of the YUTH and the CHY. Bacteriological analyses were done at YUTH Bacteriology laboratory and water filtration (membrane filtration) was done at the University of Yaoundé I Biotechnology and Environmental science laboratory. Sample collection was focused at the surgical cabinet, surgical intensive care unit, recovery room, dressing room and the surgical ward bathrooms where air, water and surface samples were collected. Surfaces included; operation table, anesthesia equipment, surgical aspirators, fan, wardrobe, office table, bedside table, vacuum cleaner, electric knife, box clamp, chair, trolley, sink, surgical blades, bed, table dressings, light box, bench, x-ray reader, clip, trays, door handle and loop. Sampling of surfaces was done by the use of sterile swabs. Trypticase soya agar was inoculated with sample, incubated at 37°C for 24-48hours. Total colonies count was done on each 16cm^2^ surface then the count density was calculated per 25cm^2^ (UFC/25Cm^2^).

For each water sample, 500 ml of water was collected in a sterile bottle containing 20 mg/l sodium thiosulfate. 100 ml of each water sample was filtered on a 0.45 mm sterile filter paper using a diaphragm vacuum pump. The filter paper was then placed on a Plate count Agar (PCA) medium, incubated at 37°C for 24-48 hours. The quantitative analysis was performed by counting all colonies on the filter paper and results were expressed in CFU/100 ml. Air samples were collected only in the area where surgical interventions were performed by spontaneous sedimentation method. In these areas, the air conditions are usually microbiologically better controlled. These samples were inoculated on Trypticase soya agar and incubated at 37°C for 24-48 hours. Morphological characteristics of bacteria colonies and biochemical methods using API20E (Biomerieux, France) were used to identify the different bacteria species. The different isolates were stored at -200°C in Brain heart infusion broth supplemented with 10% glycerol. Data analysis was done using Epi info version 3.4.3. Chi square test was used to compare categorical variables at a statistical significance level of 5%.

### Ethical Consideration

Administrative authorizations were obtained from the Director of YUTH (Pr Maurice Nkam) and from the former Director of the CHY (Pr Bella Hiag A). Ethical clearance was not applicable in the context of this study.

## Results

### Surface assessment

Of a total 143 samples in which bacterial culture were processed, 83 came from YUTH and 60 from the CHY. Bacteria was isolated in almost all samples 141 (98.60%). The average number of colonies was 132.82 CFU/25cm^2^. With regard to the classification of different hospital areas and surfaces, using the bio-contamination index values, 16 (11.18%) samples showed a reasonable number of colonies/25cm2 with 5 of these from CHY whereas 11 were from YUTH. Out of a total of 148 bacteria isolated on surfaces, 85(57.43%) were from YUTH where as 65(43.91%) were from CHY. Table 1 showed that the predominant species identified in each service was coagulase negative Staphylococcus.

**Table 1:** Distribution of isolated bacteria according to different units in each hospital

Bacteria species isolated	YUTH					CHY		YUTH & CHY	
	Operation room	Surgicale covery room	Waking room	Ward	Dressing room	Operation room	Ward	Total	%
Coagulase negative Staphylococcus	17	2	3	25	2	23	13	85	57.43
Gram positive bacillus	8	0	0	3	1	6	3	21	14.18
Staphylococcus aureus	0	1	0	3	2	5	1	12	8.10
Acinetobacter baumanii	0	2	0	2	0	3	1	8	5.40
Klebsiella pneumoniae	0	0	0	2	0	1	0	3	2.02
*Burkholderia cepacia*	1	0	0	1	0	1	0	3	2.02
Citrobacter freundii	1	0	1	0	0	2	0	4	2.70
*Flavobacterium spp*	1	0	0	1	0	0	0	2	1.35
Enterobacter cloacae	1	0	0	0	0	0	2	3	2.02
Enterobacter sakazakii	0	0	0	1	0	0	0	1	0.67
*Kingella kingae*	1	0	0	0	0	0	0	1	0.67
Escherichia coli	0	0	1	0	0	0	0	1	0.67
Providencia alcalifaciens	0	0	0	0	0	0	1	1	0.67
Sphingomonas paucimobilis	0	0	0	0	0	1	0	1	0.67
*Alcaligenes faecalis*	1	0	0	0	0	0	0	1	0.67
Chryseomonas luteola	1	0	0	0	0	0	0	1	0.67
Total	32	5	5	38	5	42	21	148	100

### Air assessment

**Figure 1:**
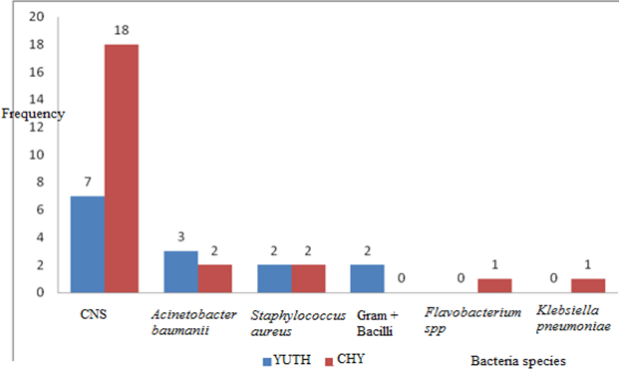
Isolated bacteria distribution from air samples according to hospital

Thirty six (36) air samples were collected and cultured, including 16 from the YUTH operation room and 20 from the CHY operation room. Thirty three (91.67%) samples were positive. A bacteria was isolated from all samples from the CHY and in 81.25% of samples from YUTH. Thirty eight (38) bacteria were identified, amongst these 14 were from YUTH and 24 were from the CHY. Frequency of identified bacteria is shown in Figure 1.

### Water assessment

Sixteen (16) water samples were collected and cultured, including 12 from the surgical service of YUTH and 4 from the surgical service of the CHY. Of the 16 cultures performed, 12 (75%) were positive. The average number of colonies was 50.93 CFU/100ml. The colony number was 22.58 CFU/100ml for samples from YUTH versus 136.00 CFU/100ml for samples from CHY. A total of 12 bacteria strains were identified, 8 from YUTH and 4 from CHY. Bacterial species identification frequency is shown in Table 2 below.

**Table 2:** Isolated bacteria species in water according to each hospital

	*Acinetobacter baumanii*	*Alcaligenes xylosoxidans*	*Burkolderia cepacia*	*Citrobacter freundii*	*Kingella kingae*	*Klebsiella Pneumoniae*	*Coagulase negative Staphylococcus*	*Serratia liquefaciens*	Total
**YUTH**	1	1	2	1	1	1	1	0	8
**CHY**	0	0	2	0	0	0	1	1	4
**Total**	1	1	4	1	1	1	2	1	12

## Discussion

The main aim of this study was to identify potential bacteria reservoirs that may be responsible for nosocomial infection amongst surgical patients at the Yaoundé University Teaching hospital and at the Central Hospital Yaoundé in Cameroon, to inform policy on the monitoring of the hospital environment in the light of control, prevention and the fight against nosocomial infections.

### 

#### Surface assessment

The findings in this study showed that the surfaces of the hospitals are heavily contaminated by bacteria with only 1.39% negative. These results are similar to those obtained by Meunier and co-workers (2005) in a study carried out in Strasbourg where they obtained à 3% negative cultures [6]. The findings also showed that the objects sampled host microorganisms. Many observations abound in the same direction and show that these objects in this environment are known to harbor microorganisms such as Staphylococcus, Enterococcus, Acinetobacter [7, 8]. The results of the colony counts suggest that microbial colonization of surfaces in these two hospitals is far higher than standard surface bio-contamination values [9]. One hundred and forty eight bacteria (148) were identified, with coagulase negative Staphylococcus (57.43%) being the most predominant species in all the surgical services. These results are similar to those obtained by Tagnouokam in 2008, who found a predominance of CNS (55%) [10]. These findings also colloborate with several studies which showed that the various components of the hospital environment (air, water, surfaces, clothes, food, medical devices, waste) can accommodate many microorganisms specifically from human or environmental origin [11, 12]. According to Kim and co-workers, (1981), the inanimate areas around patients are normally contaminated by microorganisms [13]. The presence of microbial contaminants on the surfaces is also favored by the formation of biofilms and the ability of these bacteria to survive for a long time in the environment [14].

#### Air assessment

This study showed clearly that, after performing culture and bacteria isolation on hospital air samples collected in areas where the atmosphere is under control, the level of infectious risk was acceptable in YUTH but above alert level in CHY according to the ASPEC guidelines [15]. This constitutes a significant risk factor for the occurrence of surgical site infections. These results also show that the level of aerobiocontamination at operating rooms varies from one hospital to another. Studies have shown that the air contamination was due to poor maintenance of the ventilation system or its operation disrupted by frequent opening of doors or sudden movements [16]. Moreover, the outside air naturally contains some bacterial flora in hospitals and these outside air microorganisms add to those inside and to inert tanks (water, surfaces, waste) [17]. This aero-biocontamination level observed in CHY denotes a lack of personal discipline that integrates training on hand washing and managing the operation room. Although air samples were collected during inactivity in the operation room, we isolated 25 bacteria, 65.79% of which were coagulase negative Staphylococcus. These results are comparable with those obtained in England where a gold standard method (impactation) was used. Indeed, the authors in the British study reported that among the isolated bacteria, CNS (86%) was predominant [18]. It is also important to note here that the bacteria species identified in this study are frequently identified in bacteriological analysis of the hospital environment [19]. According to the frequency of bacteria species in each of the hospitals, CNS are the most frequently isolated bacteria. This aero-biocontamination level finds its main origin in human activity. The microorganisms found in the air or on the surfaces in the operating room could be from the endogenous flora of hospital staff and patients. These suggest that the microbial environment within the operation rooms permit us to understand the possible involvement of the air in the occurrence of infections in surgical units. The works of Lidwell and co-workers (1983, 1985) have shown the relationship between the air quality in operation room and surgical wound infection occurring in prosthetic orthopedic surgery [20, 21].

#### Water assessment

Seventy five percent of water samples were culture positive. Considering the recommendations on the measurement of water biocontamination according to the ISO 19458 standard [22, 23], all the positive cultures had more than 1 CFU/100ml. This points out the fact that the use of water for standard care in these health facilities is a health risk and that ways must be identified to manage and control this risk. This result strongly suggests the role of water for standard health care in the transmission of nosocomial infections. Jebran and Mangiapan (1996); Rudnick and co-worker (1996) testify the correlation between the degree of water contamination and the occurrence of respiratory infections, mucocutaneous infections caused by Gram-negative bacteria such as *Burkholderia cepacia*, Klebsiella, Acinetobacter, Citrobacter, Serratia [24, 25]. The study by Humphrey (1989) showed that the basic gestures such as nasogastric tubes care and bathing can create aerosols and thus are sources of hospital contamination [26]. Overall, comparing the findings in this study with those of Verdeil and co-workers (1990) conducted in Toulouse France clearly suggest a significant correlation between the contamination of the hospital environment and the recovery of bacteria on surgical patients [5].

## Conclusion

The results from this study provide evidence for directly incriminating the hospital environment as a potential bacteria reservoir for nosocomial infections in the surgical patients at YUTH and CHY, thus we suggest that Public health policy makers in Cameroon must define, publish guidelines and recommendations for monitoring environmental microbiota in health facilities.
